# Targeted drug release from stable and safe ultrasound-sensitive nanocarriers

**DOI:** 10.3389/fmolb.2024.1408767

**Published:** 2024-06-19

**Authors:** Matthew G. Wilson, Aarav Parikh, Audri Dara, Alexander S. Beaver, Jan Kubanek

**Affiliations:** Department of Biomedical Engineering, University of Utah, Salt Lake City, UT, United States

**Keywords:** drug delivery, nanodroplets, nanoparticles, ultrasound, parameters, perfluorocarbon, propofol

## Abstract

Targeted delivery of medication has the promise of increasing the effectiveness and safety of current systemic drug treatments. Focused ultrasound is emerging as noninvasive and practical energy for targeted drug release. However, it has yet to be determined which nanocarriers and ultrasound parameters can provide both effective and safe release. Perfluorocarbon nanodroplets have the potential to achieve these goals, but current approaches have either been effective or safe, but not both. We found that nanocarriers with highly stable perfluorocarbon cores mediate effective drug release so long as they are activated by ultrasound of sufficiently low frequency. We demonstrate a favorable safety profile of this formulation in a non-human primate. To facilitate translation of this approach into humans, we provide an optimized method for manufacturing the nanocarriers. This study provides a recipe and release parameters for effective and safe drug release from nanoparticle carriers in the body part specified by focused ultrasonic waves.

## 1 Introduction

Pharmacological treatments are often curbed by intolerable side effects or low effectiveness of drugs (Lyons and Pahwa, 2004; Bystritsky, 2006; Zesiewicz et al., 2010; Al-Harbi, 2012; Elias et al., 2013; Elias et al., 2016; Jaffe et al., 2019). Consequently, millions of patients remain resistant to treatments and suffer from poor quality of life. There is a critical need for approaches that deliver medication selectively into the desired target in the body at a high concentration while sparing surrounding tissues.

Pioneering work in this domain utilized temperature-sensitive liposomes (Needham and Dewhirst, 2001) that can be activated by heat or radiation. However, localized heating at depth is challenging to achieve safely and in a controlled manner. Due to this issue, recent efforts have shifted to using low-intensity ultrasound as a safe and practical form of energy for localized drug release. Ultrasound can be remotely focused into a biological target at depth. Impacting circulating drug carriers, ultrasound triggers targeted release with minimal off-target effects.

Several groups have shown that ultrasound can trigger drug release from nano-sized structures stabilized with biocompatible polymeric shells (Rapoport et al., 2007; Rapoport et al., 2009; Rapoport et al., 2011; Rapoport et al., 2015; Rapoport, 2016; Airan et al., 2017; Boissenot et al., 2017; Wang et al., 2018; Wu et al., 2018; Zhong et al., 2019; Lea-Banks et al., 2021; Lea-Banks and Hynynen, 2021; Somaglino et al., 2021), and we have observed effective release in the brain of non-human primates (Wilson et al., 2024).

These nano-sized structures have been commonly filled with perfluorocarbon (PFC) cores. PFCs are highly inert and bestow the resulting nanodroplets with sensitivity to ultrasound. When exposed to ultrasound, the PFC core has been hypothesized to change phase from liquid to gas, greatly expanding in volume, and thus mediating drug release (Kripfgans et al., 2000; Sheeran et al., 2013; Doinikov et al., 2014; Shpak et al., 2014; Rapoport, 2016). Harnessing this understanding, a majority of previous studies used nanodroplets with PFC boiling points below body temperature (Rapoport et al., 2007; Rapoport et al., 2009; Rapoport et al., 2011; Sheeran et al., 2012; Rapoport et al., 2015; Rapoport, 2016; Airan et al., 2017; Wang et al., 2018; Wu et al., 2018; Zhong et al., 2019; Lea-Banks et al., 2021; Lea-Banks and Hynynen, 2021). However, successful translation of these nanocarriers has been hampered by their instability. By contrast, PFCs with high boiling points are considered to be highly safe and stable and have been well-studied for use in large quantities (Castro and Briceno, 2010; Li et al., 2018). Nonetheless, such nanodroplets have provided relatively ineffective release (Boissenot et al., 2017; Somaglino et al., 2021). In this study, we sought to identify the ultrasound parameters necessary to trigger drug release from high boiling point nanodroplets. We further sought to investigate the biocompatibility and safety of these stable nanodroplets in a nonhuman primate over a repeated dosing regimen.

In particular, this study assessed whether low-frequency ultrasound could enable drug release from stable, high boiling point drug carriers. According to the prevailing hypothesis, ultrasound delivered at the target induces vaporization of the PFC core (Sheeran et al., 2013; Doinikov et al., 2014; Shpak et al., 2014; Rapoport, 2016). This vaporization may be related to either ultrasound’s mechanical or thermal effects, which vary depending on frequency. High frequencies are more likely to engage thermal mechanisms by depositing more energy at the target. PFCs with high boiling points are unlikely to be influenced strongly by thermal effects and, thus, are expected to release drugs in response to low-frequency ultrasound more effectively. By contrast, high-frequency ultrasound, which has been used predominantly thus far, is likely to favor low boiling point PFCs. In this paper, we demonstrate that the combination of nanodroplet formulation and ultrasound parameters could provide remotely controlled, effective, and safe drug delivery. We also describe the procedure to produce the nanoparticles and release results consistently.

## 2 Results

### 2.1 *In vitro* ultrasound-triggered drug release

We prepared PFC-based, copolymer-stabilized nanodroplets loaded with the neuromodulatory drug propofol (Orser et al., 1994; Wang et al., 2018) and quantified the effectiveness of its release using an established approach in which drug is released from the nanodroplets into an organic solvent (Zhong et al., 2019). Uniquely, we tested how the critical component of the nanodroplet—its core—governs the amount of drug released as a function of ultrasound pressure and frequency. Specifically, we tested the release effectiveness of three different PFC cores—perfluoropentane (PFP), decafluoropentane (DFP), and perfluorooctylbromide (PFOB). These PFCs have boiling points of 29°C, 55°C, and 142°C, respectively. Nanodroplets with these cores had comparable sizes: mean ± SD of 543.3 ± 23.7, 550.8 ± 91.7, and 473.0 ± 28.4 nm for PFP, DFP, and PFOB, respectively.

**FIGURE 1:**
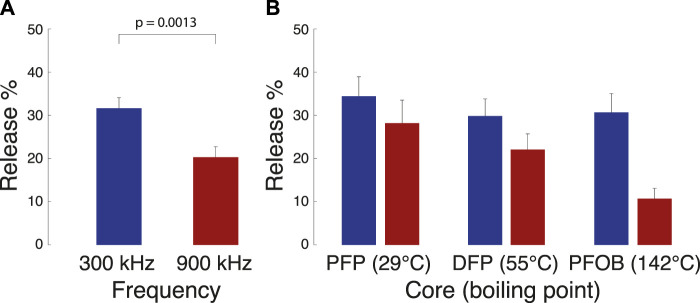
Release from nanodroplets with distinct cores under two ultrasound frequency modes. Mean ± s.e.m. propofol release relative to the amount encapsulated (see Materials and Methods for details) for the two ultrasound frequencies, combined across all cores **(A)** and the three cores tested **(B)**. The ultrasound was delivered in 100 ms pulses repeated 60 times over the period of 1 min. The *p*-value denotes the significance of a two-sample two-sided *t*-test. A complete statistical analysis of the effects is provided in Supplementary Table S1.

We used an ultrasonic transducer capable of operating at low (300 kHz) and high (900 kHz) frequency focused on vials containing the nanodroplet solutions (Zhong, 2019). We found that 300 kHz ultrasound triggered more drug release from the nanodroplets than 900 kHz (Figure 1A). The difference in the percentage of drug released at the two frequencies (31.7% and 20.3%, respectively) was significant when averaged across all cores and ultrasound pressures (*t*(130) = 3.3, *p* = 0.0013, two-sample two-tailed *t*-test). In line with our hypothesis, we found (Figure 1B) that at the higher 900 kHz frequency, the release effectiveness strongly depends on the boiling point of the PFC core—the lower the boiling point of the PFC, the higher the release effectiveness. An omnibus ANOVA model (Supplementary Table S1) that incorporated all factors tested (core, ultrasound frequency, ultrasound pressure) as well as all possible interactions, detected a significant core × frequency interaction (*F*(2, 90) = 8.05, *p* = 0.00061).

**FIGURE 2:**
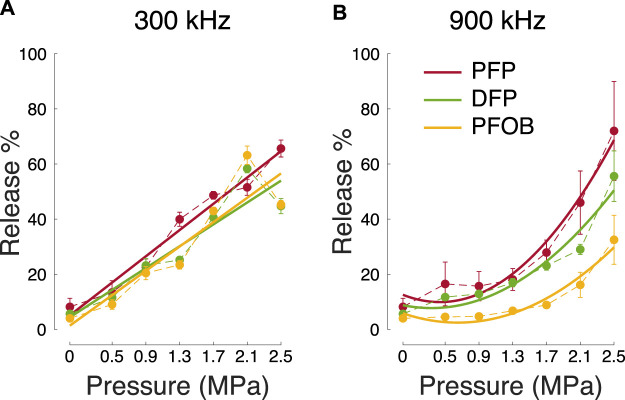
Release across all tested factors. Mean ± s.e.m. percent of the released propofol for the two ultrasound frequencies **(A)** *versus* **(B)**, ultrasound pressure amplitude (abscissa), and the three cores tested (color; see inset). The plots used *n* = 3 samples for each core and ultrasound parameter except for 0 MPa, which used *n* = 4. The thick lines represent quadratic fits to the data. For the 300 kHz data, linear fits were comparably explanatory as the quadratic fits (see text).

The scaling of the release effectiveness by the PFC boiling point (red bars in Figure 1B) suggests the engagement of a thermal mechanism at the higher frequency, in agreement with previous propositions (Zhang et al., 2010; Doinikov et al., 2014; Rapoport, 2016; Wu et al., 2018). If this is the case, the release should also scale with the average ultrasound intensity *I* delivered into the nanodroplets. For longitudinal waves, it holds $I=\frac{P^{2}}{2Z}$ (Cobbold, 2006), where *P* is the ultrasound pressure amplitude, and *Z* is the acoustic impedance of the medium. Thus, a thermal effect scales with pressure squared. We indeed found that drug release at 900 kHz showed a quadratic dependence on pressure (Figure 2B). Specifically, quadratic fits to the data (solid curves) explained 96.5, 93.5, and 94.8% of the variance in the PFP, DFP, and PFOB data points. In comparison, linear fits only explained 80.3, 80.5, and 69.3% of the variance, respectively. The difference in the mean variance explained by the quadratic and linear fits (94.9% *versus* 76.7%) was significant (*t*(2) = 4.83, *p* = 0.040, two-sided *t*-test). In contrast, the lower 300 kHz frequency showed a linear dependence of the release on pressure (Figure 2A). A quadratic fit did not significantly (*p* > 0.16) differ from the linear fit in terms of variance explained (89.5% *versus* 89.3%). The linear dependence on pressure at 300 kHz is consistent with a mechanical rather than a thermal effect (see Discussion for details).

To further validate effective release, we have complemented these *in vitro* data with *in vivo* release data in the brain of non-human primates, a topic of a dedicated study (Wilson et al., 2024). The quantity of drug released *in vivo* is dependent upon the concentration of nanodroplets present at the focus during sonication. The mean ± standard deviation amount of drug for each sample tested *in vitro* was 60 ± 20, 23 ± 11, and 5 ± 2 *μg* for PFOB, DFP, and PFP, respectively across three batches for each core.

We summarize the effects of the three factors tested (core, ultrasound frequency, and ultrasound pressure) as well as all possible interactions in an omnibus ANOVA model (Supplementary Table S1). This analysis confirms that both the core and ultrasound parameters (frequency and pressure) are significant factors for effective release. In addition, the interaction of the factors indicates that the selection of a specific core must be performed in conjunction with the appropriate ultrasound frequency.

### 2.2 Stability of PFC nanodroplets

**FIGURE 3:**
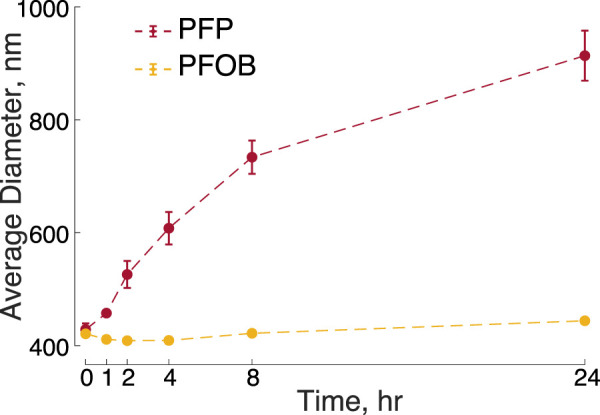
Stability of PFOB and PFP-based nanodroplets over time. Mean ± s.e.m. diameter measured at the times as indicated on the abscissa. The times were measured relative to the time of the completion of the nanodroplet production. The data include *n* = 6 samples of PFOB nanodroplets and *n* = 6 samples of PFP nanodroplets. The error bars for PFOB are smaller than the symbols.

From a safety perspective, cores with higher boiling points can be expected to be more stable, thus minimizing the risk of spontaneous drug release and embolism when injected into the bloodstream. Indeed, we found that the low-boiling point, PFP-based nanodroplets more than doubled in size over 24 h at room temperature (Figure 3, *t*(5) = 11.9, *p* < 0.001, paired two-tailed *t*-test). In contrast, the size of PFOB-based nanodroplets remained steady, increasing only by 23 nm over the 24-h period (*t*(5) = 6.3, *p* = 0.0015). A repeated measures ANOVA detected a highly significant interaction of PFC core and time (*F*(5, 50) = 43.54, *p* < 0.001), confirming that the PFP-based nanodroplets expanded at a significantly higher rate than PFOB-based nanodroplets.

### 2.3 Safety

**FIGURE 4:**
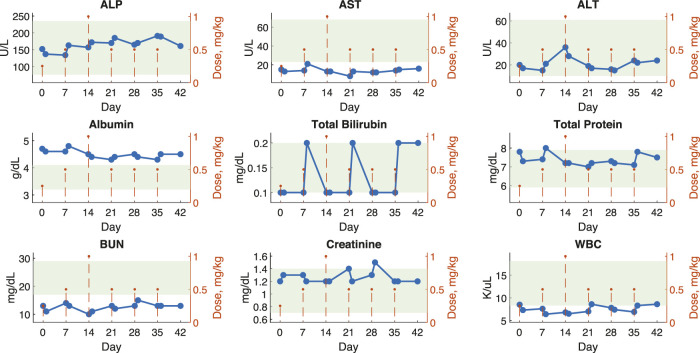
Blood metrics of safety show minimal changes following 6 weeks of continuous nanodroplet administration to a non-human primate. Blood chemistry and hematology values for a macaque monkey plotted over 42 days with 6 nanodroplet doses (orange dotted lines). Blood samples were collected 1 and 7 days after each dose (blue points). Green shaded areas represent normal values established by the Association of Primate Veterinarians. The displayed parameters are metrics of liver, kidney, and immune response function. Supplementary Figure S3 shows all other parameters.

We have evaluated the safety of the PFOB-based nanodroplets in a non-human primate. To evaluate the safety of repeated dosing of PFOB nanodroplets, we completed a series of blood draws to monitor clinical chemistry and hematology parameters. A total of six doses of nanodroplets were administered: an initial ramp-up at 0.25, 0.5, and 1.0 mg/kg of propofol followed by three additional doses at 0.5 mg/kg, which we have previously found to elicit robust effects on choice behavior of non-human primates (Wilson et al., 2024). This approach provides detailed insights into the potential toxicity of the nanocarriers to the liver (ALP, AST, ALT, total protein, albumin, and bilirubin), kidneys (creatinine, BUN), or spleen (RBC, WBC, and platelet counts) where these nanodroplets are expected to accumulate (Airan et al., 2017; Wilson et al., 2024). We also assessed the level of immune system activation using the WBC count. These key indicators along with the normal range for rhesus macaques are shown in Figure 4. All other blood chemistry and hematology markers are shown in Supplementary Figure S3. To evaluate both short-term and long-term effects, we quantified changes in these parameters 1 and 7 days after each nanodroplet dose at or above 0.5 mg/kg (Supplementary Figure S4). Across these comparisons, only blood glucose showed a significan difference (*t* (4) = 6.03, *p* = 0.004; one-sample *t*-test), decreasing on average by 21 mg/dL 1 day following the injection. The results associated with the individual *t*-test as well as 95% confidence intervals are shown in Supplementary Table S3.

### 2.4 A versatile manufacturing process for ultrasound-responsive nanocarriers

**FIGURE 5:**
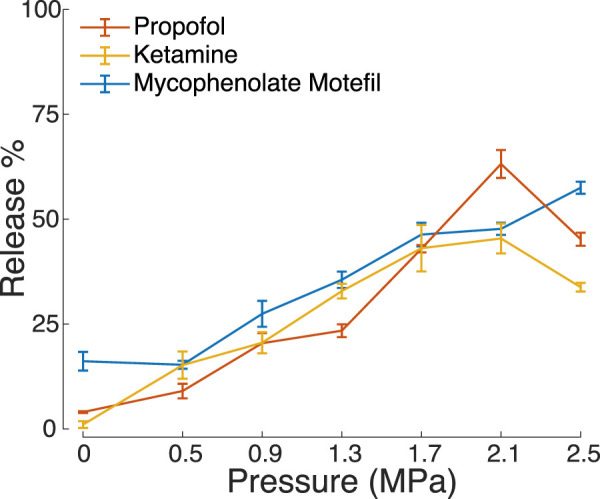
Hydrophobic drugs encapsulated and released by PFOB nanodroplets. Mean ± s.e.m. percent drug released under ultrasound stimulation under the same experimental conditions as in Figure 2.

To demonstrate the versatility of the PFOB nanodroplets, we loaded them also with ketamine (an anesthetic with longer effect duration) and mycophenolate motefil (an immunosuppressant). We observed a similar sensitivity to ultrasound as the propofol-loaded nanodroplets (Figure 5). Indeed, an ANOVA model incorporating ultrasound pressure and drug type indicated no significant effect of drug type (*F*(1, 46) = 0.88, *p* = 0.4). Mycophenolate motefil-loaded nanodroplets did release more drug without ultrasound (*t*(5) = 6.5, *p* = 0.0013, two-sample two-tailed *t*-test). This may be attributed to the lower hydrophobicity compared to propofol and ketamine, leading to less effective encapsulation inside the nanodroplets.

**FIGURE 6:**
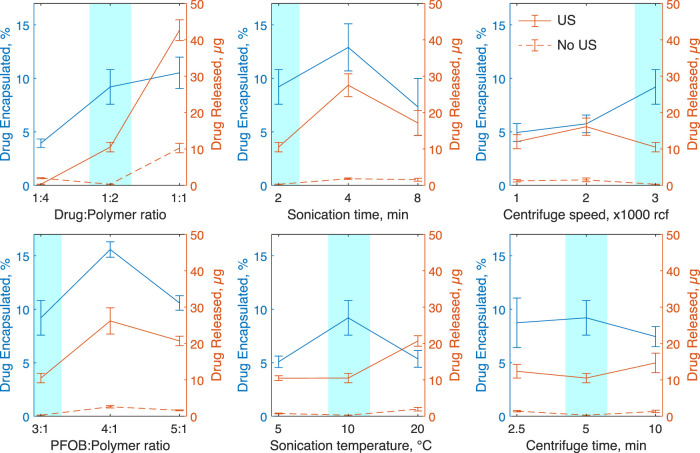
The effects of nanoparticle manufacturing parameters on drug encapsulation and release. Each plot shows the mean ± s.e.m. propofol encapsulated as a percentage of propofol used in blue and total drug released with (solid line) and without ultrasound (dotted line). In these tests, 300 kHz ultrasound was applied for 60 s in 10 ms bursts at a rate of 10 Hz and peak negative pressure of 1.5 MPa. Each parameter test involved five independent nanoparticle samples. Baseline parameters highlighted in blue were held constant while other parameters were varied.

To provide the reader with a recipe to reliably manufacture the nanoparticles, we systematically studied key variables in the manufacturing process. This included the ratio of nanodroplet components (drug and PFOB to polymer), sonication bath parameters (temperature and duration), and centrifuge parameters (speed and duration). The results are summarized in Figure 6, which shows how each variable affects drug encapsulation and release. Supplementary Table S2 summarizes the statistical analysis of associated with this evaluation. The table indicates which manufacturing parameters significantly affect the nanodroplet size, drug encapsulation, and release.

These data show that the ratios of polymer shell to drug and PFOB core strongly influence the resulting nanodroplet composition, and thus must be selected carefully to meet specific application criteria. For example, increasing the ratio of drug to polymer shell significantly increases the rate of encapsulation and drug release, but also increases the rate of spontaneous drug release and the average particle size (a change from 780 ± 80 nm to 3300 ± 600 nm in size). A full summary of size results can be found in Supplementary Figure S1. We found no significant linear correlation between particle size and encapsulation (*p* = 0.61) or particle size and drug release with (*p* = 0.47) or without ultrasound (*p* = 0.89) (Supplementary Figure S2).

## 3 Discussion

This study investigated the effectiveness and safety of drug release from biocompatible nanodroplets triggered by focused ultrasound. Uniquely, we evaluated the release efficacy from nanodroplets filled with PFCs with distinct boiling points, including boiling points above the body temperature, and investigated the relevant ultrasound parameters that activate them. We found that low frequency ultrasound (300 kHz in particular) was more effective at mediating release in general and that low frequencies are paramount for effective release from PFCs with a high boiling point.

The core has been hypothesized to be a critical factor in governing the effectiveness of ultrasound-based drug release (Rapoport et al., 2011; Wu et al., 2018; Somaglino et al., 2021). Indeed, our 900 kHz data show (Figure 1B, red) that at the range of commonly applied ultrasound frequencies (about 1 MHz and greater), the core is a key determinant of release effectiveness. We found that the higher the boiling point of the encapsulated PFC, the lower the release effectiveness. This finding is in accord with previous results (Zhang et al., 2010; Doinikov et al., 2014; Rapoport, 2016; Wu et al., 2018). Critically, we found that lowering the frequency to 300 kHz increased the release effectiveness at a general, core-independent level (Figure 1). This effect can also be found in a study that used PFP (Zhong et al., 2019). The overall effect of decreasing frequency may be enhanced by the increase in focal volume, but the differences between cores at each frequency remain informative. The application of low-frequency ultrasound thus opens the path to using high boiling point cores (e.g., PFOB) as release actuators. A frequency of 300 kHz provides spatial resolution on the order of several millimeters, which approximates the focal volume of the drug released in tissue by ultrasound (Wang et al., 2018; Lea-Banks and Hynynen, 2021). While the focal size is larger than ultrasound of higher frequencies, the focal size remains applicable for confined targets, including in the brain (Weniger et al., 2006; Bette et al., 2018).

This work uniquely evaluated the safety of ultrasound responsive PFC nanodroplets over 6 weeks of repeated dosing in a nonhuman primate. This type of dosing regime may be necessary for long-term treatments. The minimal impact on measures of toxicity in a nonhuman primate over this time period are a key indicator of potential for clinical translation. Indeed, only blood glucose was found to show a statistically significant change at 1 day after the nanodroplet administration. The level stabilized at day 7. This change in blood glucose may be attributed to diet—the monkey received sweet juice as a reward on the days of the nanodroplet administration. Since we did not fast the animals in these experiments, we could not reliably use the blood glucose level as a metric of pancreatic inflammation (Sun et al., 2019). Without fasting, most of the circulating glucose is attributed to diet (McMillin, 1990).

This longer-term analysis aligns with our previous work, which quantified the immediate response to nanodroplet administration (Wilson et al., 2024). That study found a modest and transient increase in white blood cell count. The increase was within the normal range. We observed no significant change in white blood cell count in this study, which indicates that any immune system activation resolves within the first day following administration.

The majority of studies that applied ultrasound to PFC-based nanodroplets used PFCs with boiling point below the body temperature (Rapoport et al., 2007; Rapoport et al., 2009; Rapoport et al., 2011; Sheeran et al., 2012; Rapoport et al., 2015; Rapoport, 2016; Airan et al., 2017; Wang et al., 2018; Wu et al., 2018; Zhong et al., 2019; Lea-Banks et al., 2021; Lea-Banks and Hynynen, 2021). Although the Laplace pressure of sub-micron particles can effectively increase the boiling point when encapsulated (Rapoport, 2012), PFCs with low boiling points may suffer from instability issues, which has raised safety concerns. Indeed, our data show that PFP-based nanodroplets not exposed to ultrasound spontaneously increase in size over a short time window, whereas PFOB nanodroplets remain stable (Figure 3). The increase in size can be attributed to either Ostwald ripening or droplet vaporization. Ostwald ripening in emulsions is the process of larger droplets becoming larger by diffusion of the PFC from smaller droplets. This effect is diminished for higher molecular weights (Kabalnov and Shchukin, 1992). This applies to our findings—PFOB has a molecular weight of 498.97 g/mol while PFP is only 288.04 g/mol. Spontaneous vaporization of the PFP core may also be at play, since the boiling point of PFP is close to room temperature. Further, low boiling point PFCs can form persistent microbubbles after vaporization by ultrasound (Rapoport et al., 2009; Rapoport, 2016; Lea-Banks et al., 2021). A combination of these effects may also be at play in the observed increase in size: Ostwald ripening may lower the vaporization threshold of the PFC core by reducing the Laplace pressure. These data suggest that PFP nanodroplets, if at all, should be used immediately following their production, or else be stabilized, as accomplished previously by freezing with added glycerin (Wang et al., 2018). It is nonetheless unclear if PFP nanodroplets would expand more quickly than they are cleared from the bloodstream *in vivo*; more work is required on this matter. Our finding that PFOB can be used for effective release from nanodroplets at low ultrasound frequencies (Figure 1; Figure 2) should mitigate the boiling-point associated concerns. Indeed, PFOB-based products including LiquiVent—an oxygen-carrying liquid drug, and Oxygen (both Alliance Pharmaceutical Corporation, San Diego, CA, United States of America)—a blood substitution agent (Cohn and Cushing, 2009; Castro and Briceno, 2010; Li et al., 2018) have been thoroughly studied for use in liter-quantities.

Perfluorocarbons are cleared via the reticuloendothelial system (RES), primarily by macrophage phagocytosis, and ultimately eliminated via exhalation from the lungs (Flaim, 1994). The clearance half-life of PFOB is estimated to be 3 days (Riess, 1988; Flaim, 1994). In clinical trials, side effects are typically transient and primarily associated with activation of macrophages at doses much higher than used here (Flaim, 1994; Flaim et al., 1994). PFCs have been studied at doses starting around 2 g/kg for blood substitution applications, while our maximum dose was only 0.14 g/kg.

The *in vitro* release data presented here also contribute to the understanding of the release mechanism. Thus far, the predominant hypothesis of action has been the vaporization of the PFC droplet upon the impact of focused ultrasound (Rapoport et al., 2011; Sheeran et al., 2013; Doinikov et al., 2014; Shpak et al., 2014; Wu et al., 2018; Somaglino et al., 2021). In this mechanism, the thermal and mechanical aspects of propagating ultrasound exceed the vaporization threshold governed by the PFC boiling point and the Laplace pressure. The PFC core increases in size (up to a 5-fold increase in diameter upon vaporization (Rapoport, 2012)). This increase in diameter is expected to increase the rate of drug diffusion through the polymer shell, contributing, in addition to any superimposed effects of ultrasound, to drug release. Indeed, our data at the common range of ultrasound frequencies, 900 kHz, provide two lines of evidence in support of this hypothesis. First, we found that the release increases with decreasing boiling point (Figure 1B), as expected from the vaporization hypothesis. Second, thermal energy delivered by ultrasound is known to be proportional to pressure squared (Cobbold, 2006). We indeed found a quadratic dependence of the release on pressure (Figure 2B).

Our and a previous study (Zhong et al., 2019) suggest that ultrasound of frequencies lower than those in the common diagnostic range may mediate more effective drug release. Lower frequencies are known to accentuate mechanical effects, which can take two forms. First, ultrasound can induce cavitation, the formation of gaseous nuclei from dissolved gasses under the low-pressure epochs of the ultrasound pressure wave (Apfel and Holland, 1991). Cavitation can provide useful mechanical forces until it exceeds a threshold at which the formed gaseous nuclei collapse and cause damage (Apfel and Holland, 1991). From the FDA’s 510(k) Track 3 standpoint (FDA, 2019), cavitation is unlikely to occur for mechanical index—defined as ultrasound pressure divided by the square root of frequency—values below 1.9. In our hands, a 300 kHz, 1.0 MPa pressure at target yields a mechanical index of 1.83. Despite being below the value of 1.9, this pressure level already drives significant release (Figure 2A). Thus, if cavitation is involved, it likely constitutes one of multiple mechanisms. Another candidate mechanism for drug release is particle displacement. The maximal displacement of a particle in the ultrasound path, *ξ*_*m*_, is linearly proportional to the ultrasound pressure amplitude *P*: $\xi_{m}=\frac{P}{Z}\int_{0}^{T/2}\sin\left(2\pi Ft\right)dt=\frac{P}{Z\pi F}$ , where *F* is the ultrasound frequency and *Z* the acoustic impedance. Indeed, our 300 kHz data show a linear dependence of the release on pressure (Figure 2A). This mechanism is supported by high-speed imaging, which did not detect a persistent phase change despite effective release in rodents (Zhong et al., 2019). Together, our findings indicate that both thermal and mechanical effects can be at play, depending on the applied ultrasound frequency and the PFC core selected. Higher frequencies accentuate thermal effects (PFC vaporization) and require low-boiling point cores, whereas lower frequencies accentuate mechanical effects (cavitation or cycle-by-cycle particle displacement) and enable the use of stable, high-boiling point cores.

The mechanical nature of the drug release effect at low frequencies is beneficial for clinical translation, as no heating of the target tissue is required. Indeed, we have observed no signs of damage in diagnostic MRI at the ultrasound target with 30 ms ultrasound pulses at 1.5 MPa in nonhuman primates (Wilson et al., 2024). To minimize the potential for heating, clinical studies may use shorter pulses and lower duty cycles, which decreases the deposited energy. The risk of heating can also be reduced using an approach that corrects for the skull and hair to deliver a predictable dose of ultrasound (Riis et al., 2023; Riis et al., 2024). To further reduce the risk of unwanted heating in clinical studies, MR thermometry could be used to monitor for potential temperature rise at the focus and throughout the brain. Cavitation detection systems have also be used to identify the minimum threshold to trigger drug release (Lea-Banks and Hynynen, 2021), thus minimizing the delivered energy.

Interestingly, at 300 kHz, we observed a marginal decrease in drug release from DFP and PFOB nanodroplets at the highest ultrasound pressure tested (Figure 2A). This effect has been also observed in another study (Lea-Banks and Hynynen, 2021). These high pressures may lead to cavitation—the formation of bubbles—which may consequently block the propagation of ultrasound into the target (Lea-Banks and Hynynen, 2021). PFP did not exhibit this effect, possibly due to the lower boiling point—complete vaporization and subsequent collapse of PFP bubbles may prevent shielding effects with the repeated pulsing scheme used here.

The study has four limitations. First, we tested the effects of ultrasound frequency and pressure, but not the ultrasound waveform. For instance, we have not tested the effects of the duty cycle and only used a fixed value of 10%. Increases in the duty cycle are known to lead to stronger, cumulative effects (Airan et al., 2017; Zhong et al., 2019). Optimizing the ultrasound parameters would help to maximize safety and efficacy of drug release by avoiding unneeded increases in ultrasound pressure or duration. For example, pulse duration has been evaluated systematically, indicating that pulses longer than 50 ms reach diminishing returns (Airan et al., 2017). Second, we quantified the release of nanodroplets using a single copolymer shell (PEG:PDLLA). This limitation is mitigated by a previous study that found little variability in the release due to specific forms of the polymeric shell (Zhong et al., 2019). However, different polymer shells may change the interactions of the nanodroplets and the immune system. This has substantial implications on the drug released *in vivo*. For example, longer PEG chains have been used to extend blood circulation time (Pata et al., 2003). Further, while nanodroplet size did not affect drug release or encapsulation rates in this study, it may affect the pharmacokinetics. Future work *in vivo* could help optimize the composition of these droplets. Third, the selection of parameters for the drug release study limits our ability to make definitive conclusions about the mechanism of action. As mentioned previously, we uncovered the involvement of a mechanical effect in the drug release from high-boiling point PFCs, but whether the effect is attributed to cavitation, particle displacement, or another mechanism remains unclear. Studies of the acoustic emissions from the nanodroplets could quantify the likelihood of cavitation (Xu et al., 2020; Welch et al., 2022), and high-speed imaging studies could identify the presence of vaporized droplets (Sheeran et al., 2013; Doinikov et al., 2014; Puett et al., 2014). Finally, the *in vitro* drug release assay does not fully replicate the conditions *in vivo*, which we have evaluated in a dedicated study (Wilson et al., 2024). Blood contains many proteins and cells which may interfere with the function of the nanodroplets or diffusion of drug after activation, and uptake into brain tissue may differ from uptake into hexane. The pharmacokinetics of the nanodroplets are also a critical *in vivo* factor that was not addressed here. However, past studies by our group (Wilson et al., 2024) and others (Wang et al., 2018; Lea-Banks et al., 2020) have found a half-life of several minutes in circulation, which has allowed sufficient drug release to modulate behavior (Lea-Banks et al., 2020; Wilson et al., 2024). Additional studies have mapped the extent of drug release in the brain from PFC nanodroplets, indicating a precisely targeted region of release (Lea-Banks and Hynynen, 2021). Nonetheless, further work will be needed to accurately predict the amount of drug release at the target. While the absolute values for drug release presented here may not translate directly to *in vivo* situations, we have emphasized the relative comparisons between nanodroplet compositions and the ultrasound frequencies.

## 4 Conclusion

In summary, we found that low-frequency ultrasound can effectively release drugs from stable PFC-based nanocarriers. This study informs the use of specific PFC-based nanodroplets and ultrasound parameters for effective, safe, and targeted drug release in humans while providing evidence of biocompatibility in nonhuman primates over an extended period of time. To facilitate the translation into applications in humans, we report the parameters critical for robust assembly and production of the nanoparticles. Further work is still needed to more accurately predict or measure the drug dose administered at target *in vivo*, which depends significantly on the quantity of nanodroplets present at the target. Specifically, the field would benefit from more detailed pharmacokinetics studies and real-time feedback systems for nanodroplet concentration and drug release effectiveness. This approach has a variety of clinical applications depending on the drug loaded and the target region. A short-lasting psychoactive drug such as propofol could be used to systematically modulate specific parts of the brain in a diagnostic capacity, while longer-lasting drugs such as ketamine could then induce neuroplastic changes. Outside of neuromodulation, there are many applications which would benefit from localized release of drugs, including glioblastoma where localized release of chemotherapeutics could reduce the overall dose required, or organ transplants, where immunosuppresants could be localized to the transplanted organ. The identification of stable drug carriers and compatible ultrasound parameters for effective release provided here lower the barrier for clinical translation of these approaches.

## 5 Materials and methods

### 5.1 Materials

Methoxy poly(ethylene glycol)-b-poly(D,L-lactide) (PEG-PDLLA) co-polymers with 2,000 : 2,200 g/mol molecular weights, respectively, were obtained from PolyScitech (United States of America). 2H-, 3H- decafluoropentane and perfluorooctyl bromide were obtained from Tokyo Chemical Industry Co. (Japan). Perfluoro-n-pentane was obtained from Strem Chemicals (United States of America). Propofol was obtained from Sigma Aldrich (Millipore Sigma, Canada). Infrared dye IR800RS NHS Ester was obtained from LI-COR Biosciences (United States of America). HPLC-grade tetrahydrofuran (THF) and methanol were obtained from Fisher Scientific (United States of America). Phosphate buffer solution (PBS) was obtained from Gibco (Thermo Fisher Scientific, United States of America) and Cytiva (United States of America). Mycophenolate Motefil was obtained from Akorn, Inc (United States of America). Ketamine was obtained from VetOne (United States of America).

### 5.2 Nanodroplet production

**FIGURE 7:**
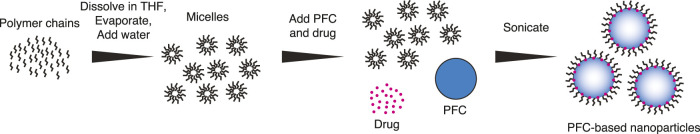
Nanodroplet production. The conversion of polymeric micelles into PFC-core-based nanodroplets using ultrasound. The three steps are described in detail in the text.

The process of manufacturing the drug-encapsulating, ultrasound-responsive PFC particles is illustrated at a conceptual level in Figure 7 and described in detail in previous studies (Rapoport et al., 2007; Zhong et al., 2019). The process converts small ( <30 nm) micelles into much larger ( >300 nm) PFC-filled nanodroplets. First, the PEG-PDLLA polymer constituting the basis of the nanodroplet shell is dissolved in THF at a rate of 1 mL THF: 16 mg polymer. For the biodistribution and blood clearance studies, infrared dye is added at a ratio of 1:32 (dye:polymer) for the rats and marmoset and 1:110 or 1:89 for the macaques 1 and 2, respectively. THF is then evaporated under vacuum until a gel-like layer remains. PBS is added at a rate of 1 mL PBS: 8 mg polymer and placed on a shaker table at 120 rpm to dissolve for 15 min. The addition of PBS orients the hydrophilic copolymer, PEG, toward the water and the hydrophobic, PDLLA, copolymer away from the water, and as a consequence, micelles are formed. Next, the PFC core and propofol are added and emulsified. A ratio of 1 mg propofol: 2 mg polymer was used in all cases. The nanodroplets’ diameter can be controlled by the ratio of PFC to polymer, as reported previously (Rapoport et al., 2011). For PFOB and DFP nanodroplets, a ratio of 4.5 *μ*L PFC: 1 mg polymer was used. The ratio for PFP was scaled up to 6.25 *μ*L: 1 mg to account for PFC lost to vaporization before being emulsified. A 20 kHz, 500 W sonicator with a cup horn attachment (VCX500, Sonics) was used to perturb the thermodynamic equilibrium of the micellar system, which leads to the incorporation of PFOB into the micelles and the formation of stable nanodroplets or nanodroplets (Gupta et al., 2015). The PFC and drug are added to 15 mL centrifuge tubes and gently shaken to combine before adding 8 mL of the micelle solution. The samples are then sonicated in a cold bath at 20% power in 30-s intervals until the solution is cloudy and drug and PFC are fully emulsified (1–3 min in total). A custom temperature-controlled cooling system maintained the bath temperature during sonication at 2°C for PFP and 10°C for DFP and PFOB. PFP must be kept colder to minimize vaporization before emulsification, while DFP and PFOB require higher temperatures to emulsify successfully without vaporizing. We found this controlled temperature approach to maximize the consistency of the nanodroplet sizes, drug encapsulation, and release properties. The resulting solution contains the desired nanodroplets in addition to remaining micelles, dissolved polymer, and free propofol. Nanodroplets are isolated using three cycles of centrifugation at 3,000 relative centrifugal force (RCF) at 4°C. After each cycle, the supernatant is discarded and the pellet dissolved in 5 mL fresh PBS. If the resulting solution contains larger particles than needed, these were removed by a slower centrifuge cycle for 1 min at 800 RCF, this time keeping the supernatant. Larger particles contained in the pellet are discarded.

### 5.3 Hydrophobic drug preparation

The propofol used was already in its hydrophobic form, but mycophenolate motefil and ketamine had to be modified from hydrochloride salts to be encapsulated in the nanodroplets. To do so, mycophenolate motefil was first dissolved in deionized water in a centrifuge tube at a concentration of 10 mg/mL. Ketamine was obtained in solution at a concentration of 100 mg/mL. Then, 3N NaOH was added dropwise at an equal molar ratio with the HCl until the drug precipitate forms. The solution was then centrifuged for 5 min at 600 RCF and the supernatant discarded. Centrifugation was repeated for a total of three cycles, resuspending in deionized water after each time. After the final centrifuge cycle, the drug was dissolved in methanol then filtered through a 0.2 micron syringe filter. Methanol was then dried under a gentle nitrogen stream, leaving the hydrophobic drug in a powdered form which could be incorporated into nanodroplets.

### 5.4 Nanodroplet characterization

The sizes were measured using a Zetasizer Nano S (Malvern Panalytical, United Kingdom), which reports the intensity-weighted size distribution. The size values reported in the Results section describe the mean ± standard deviation of the distribution of the intensity values measured by the device. To quantify the amount of drug encapsulated, a 50 *μ*L solution of nanodroplets is added to 450 *μ*L of methanol to dissolve all components (Airan et al., 2017). A UV-Vis spectrophotometer (NanoDrop 2000; Thermo Scientific) is used to quantify the concentration by comparing the absorbance to a standard curve at 276 nm for propofol, 268 nm for ketamine, and 305 nm for mycophenolate motefil.

### 5.5 Apparatus

As in a previous study (Zhong et al., 2019), drug release is quantified in standard 1.5 mL microcentrifuge tubes. Each tube with freshly produced nanodroplets is placed into a plastic holder. A focused ultrasonic transducer (H-115, 64 mm diameter, 52 mm focal depth, Sonic Concepts) was positioned 52 mm below the holder so the sample was within the ultrasound focus. Degassed water (AIMS III system with AQUAS-10 Water Conditioner, Onda) mediated coupling between the ultrasound face and the vial. The transducer was operated at 300 kHz and the third harmonic, 900 kHz. Stimuli were generated using a function generator (33520b, Keysight, United States). The signals were amplified using a 55-dB, 300 kHz–30 MHz power amplifier (A150, Electronics & Innovation, United States).

### 5.6 Ultrasound parameters

The ultrasound carrier frequencies for *in vitro* experiments were 300 kHz and 900 kHz. For the assessment of different PFC cores and drug encapsulated, continuous pulses 100 ms in duration were repeated once per second for a total of 60 s (Airan et al., 2017; Zhong et al., 2019). The pressure levels at the vial location, measured in degassed water, were 0, 0.5, 0.9, 1.3, 1.7, 2.1, and 2.5 MPa. During the nanodroplet manufacturing study, ultrasound was applied in 10 ms pulses 10 times per second for 1 minute. The pressure fields were measured using a capsule hydrophone (HGL-0200, Onda) calibrated between 250 kHz and 40 MHz and secured to 3-degree-of-freedom programmable translation system (Aims III, Onda).

### 5.7 Drug release characterization

100 *μ*L of hexane was placed on top of 200 *μ*L nanodroplet solutions prior to sonication to act as a sink for released drug (Airan et al., 2017). After 1 min, 45 s of total incubation time, 50 uL of hexane was extracted. The amount of dissolved propofol was quantified using UV-Vis spectrophotometry as described previously. The percent release efficacy is defined as the amount of the drug released into the hexane relative to the amount encapsulated. Each datapoint in Figure 2 included 3-4 distinct samples.

### 5.8 Nonhuman primate biocompatibility

Biocompatibility was tested in one rhesus macaque (*macaca mulatta*, male, age 10 years, weight 14 kg). Nanodroplets were administered intravenously once per week using vascular access ports implanted in the right saphenous vein (Graham et al., 2010). Nanodroplets were manufactured as described above and doses quantified by the concentration of propofol. The dose was ramped up gradually: 0.25 mg/kg, 0.5 mg/kg, and 1.0 mg/kg to detect potential dose-dependent effects. We then reduced the dose to 0.5 mg/kg for three additional sessions as we found this level to induce robust behavior effects in a previous study (Wilson et al., 2024). 2 mL of blood was drawn before the first dose then 1 and 7 days after each dose. Samples were analyzed by IDEXX and normal ranges were provided by the Association of Primate Veterinarians (green areas in Figure 4; Supplementary Figure S3). To quantify any potential effects, the baseline for each dose was subtracted from the measurements at day 1 and 7. The lowest dose was excluded from this analysis because its efficacy is unknown. To detect effects, we ran two-tailed, one-sample t-tests for each parameter at days 1 and 7 and computed a 95% confidence interval for each result (Supplementary Table S3).

## Data Availability

The raw data supporting the conclusion of this article will be made available by the authors, without undue reservation.
