# Supplementary material for: Targeted drug release from stable and safe ultrasound-sensitive nanocarriers
Source: Front Mol Biosci. 2024 Jun 19;11:1408767. doi: 10.3389/fmolb.2024.1408767 (PMC11219560; doi:10.3389/fmolb.2024.1408767)
Supplement: Supplementary file 1 [file DataSheet1.pdf]

SUPPLEMENTARY MATERIAL

Factor	Significance
Core	**< 0.001**
F	**< 0.001**
P	**< 0.001**
Core × F	**< 0.001**
$F \times P$	**< 0.001**
Core × P	**0.030**
Core × $F \times P$	**0.049**

Table S1. Summary of nanodroplet core and ultrasound effects. The effects of the nanodroplet core, ultrasound frequency (F), and ultrasound pressure (P). These effects were assessed using a three-way ANOVA that featured the three main effects and all possible interactions. Bold entries are significant ($p < 0.05$).

	Significance			
Factor	Encapsulation	Release US	Release no US	Size
Drug:polymer ratio	**0.008**	**< 0.001**	**< 0.001**	**< 0.001**
PFOB:polymer ratio	**0.004**	**0.002**	**< 0.001**	**0.015**
Sonication time	0.232	**0.004**	**0.003**	0.070
Sonication temperature	**0.035**	**< 0.001**	**0.005**	0.514
Centrifuge time	0.052	0.144	0.074	**0.011**
Centrifuge speed	0.761	0.379	**0.008**	**0.026**

Table S2. Summary of manufacturing parameters. The effects of nanodroplet manufacturing parameters assessed using a one-way ANOVA for each factor and metric tested. Bold entries are significant ($p < 0.05$).

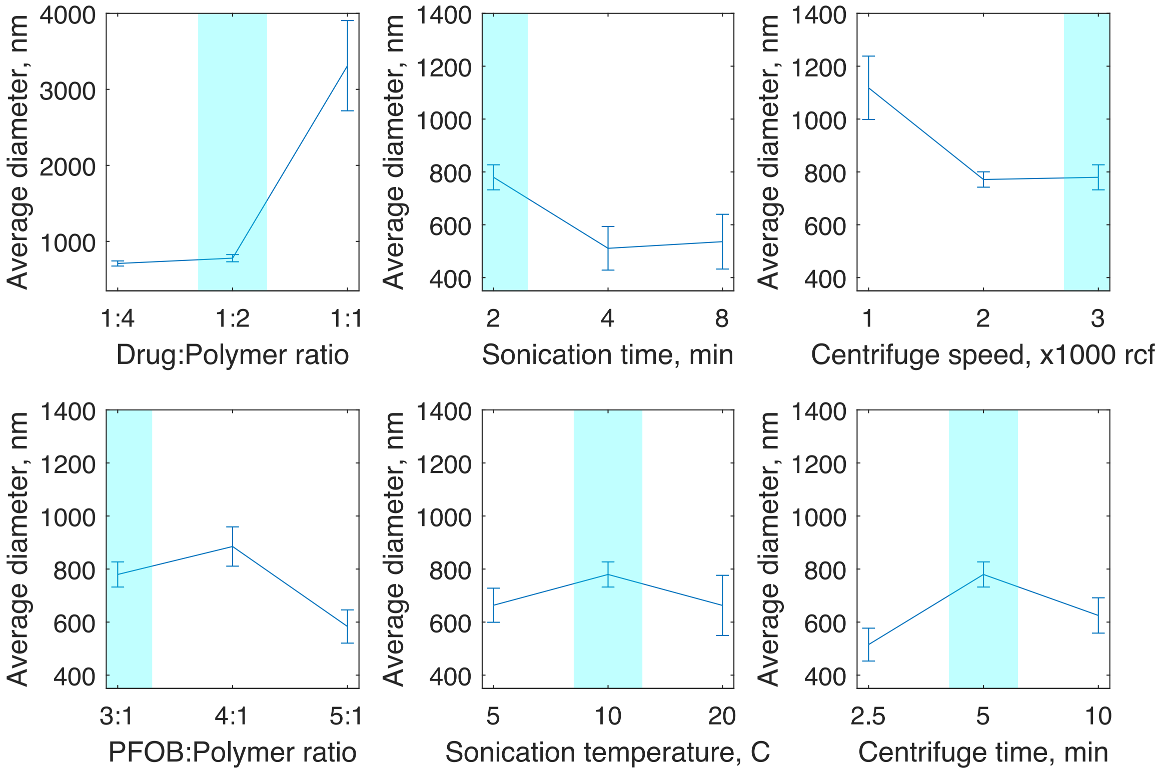

Figure S1. Effects of manufacturing parameters on particle size. Mean±s.e.m. average diameter of nanodroplet samples for each manufacturing method.

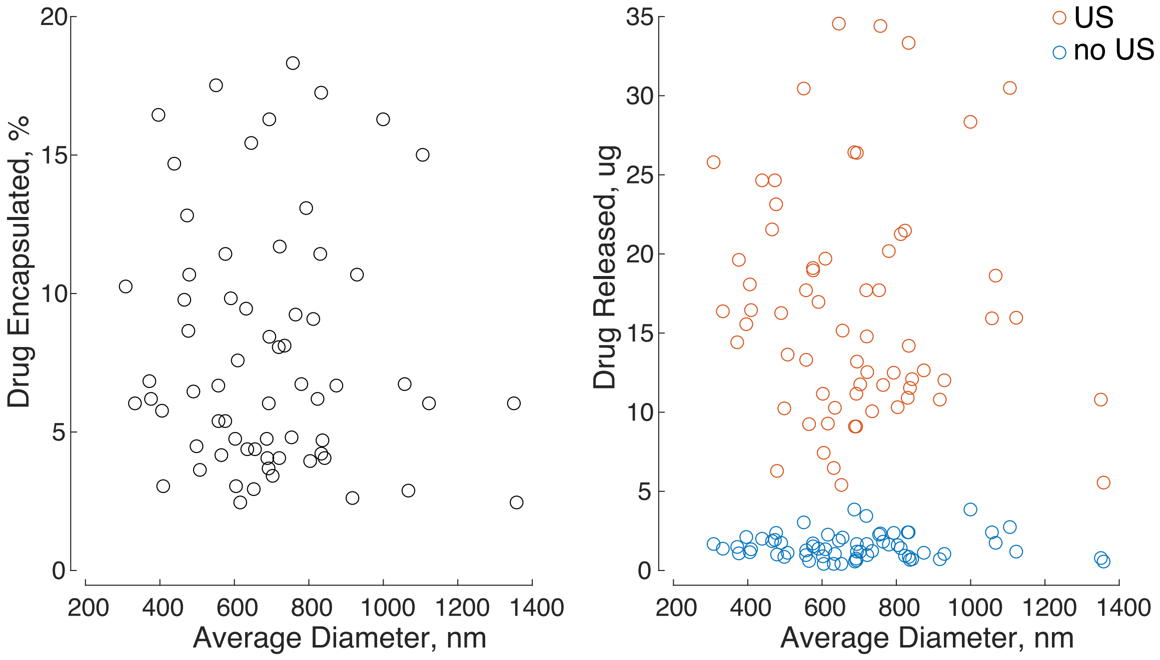

Figure S2. Particle size effects on drug encapsulation and release. Left: Drug encapsulation in nanodroplets as a percentage of drug used for each sample in the manufacturing experiments. There is no significant correlation between average particle size and drug encapsulated. Right: Drug released from each nanodroplet sample with (orange) and without (blue) ultrasound (US). There was no correlation between particle size and drug release either with or without US.

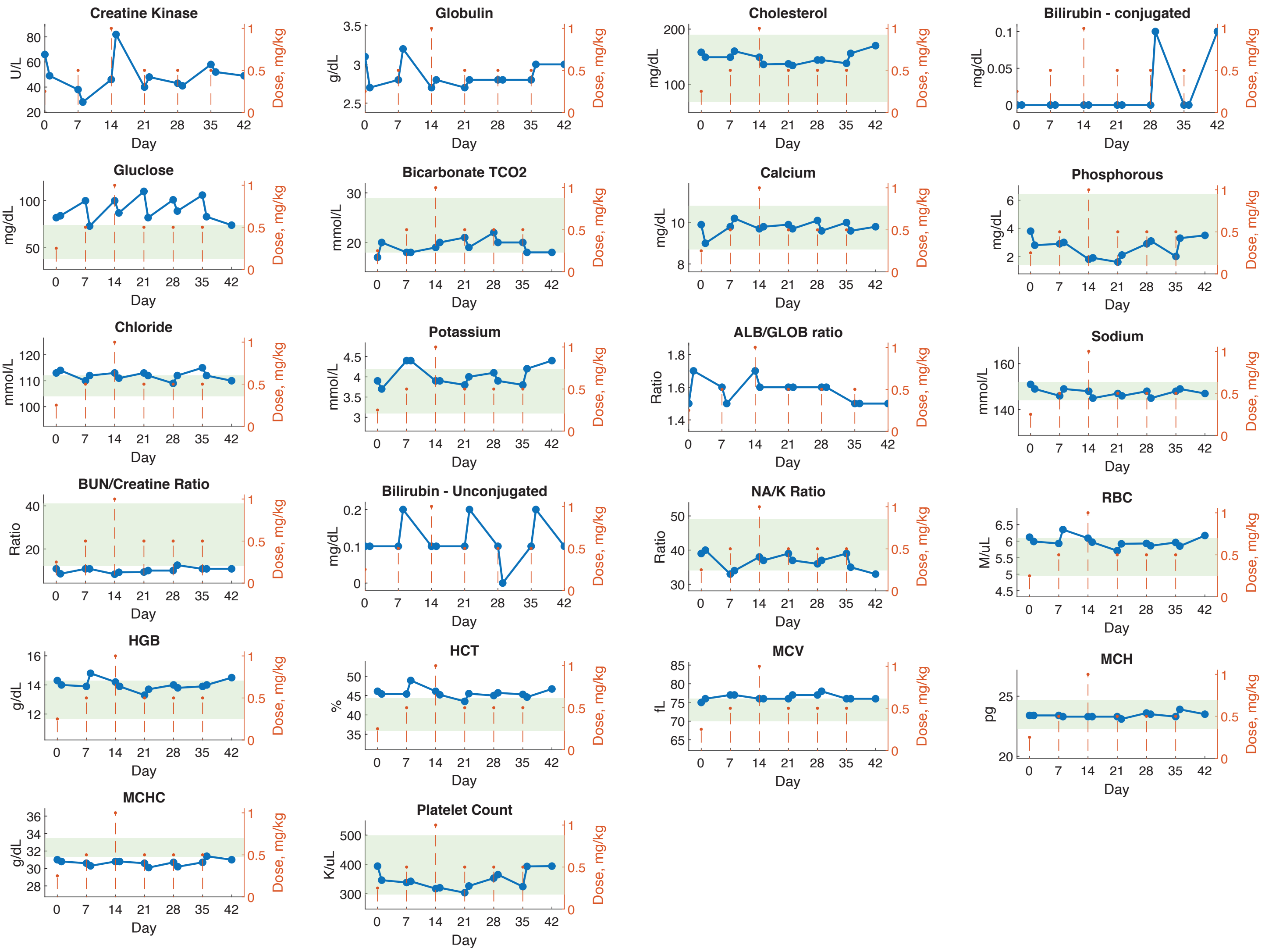

Figure S3. Blood chemistry and hematology from 6-week nanodroplet dosing. Blood chemistry and hematology values for one macaque monkey plotted over 42 days with 6 nanodroplet doses (orange dotted lines). Blood samples were collected one and 7 days after each dose (blue points). Green shaded areas represent normal values established by the Association of Primate Veterinarians.

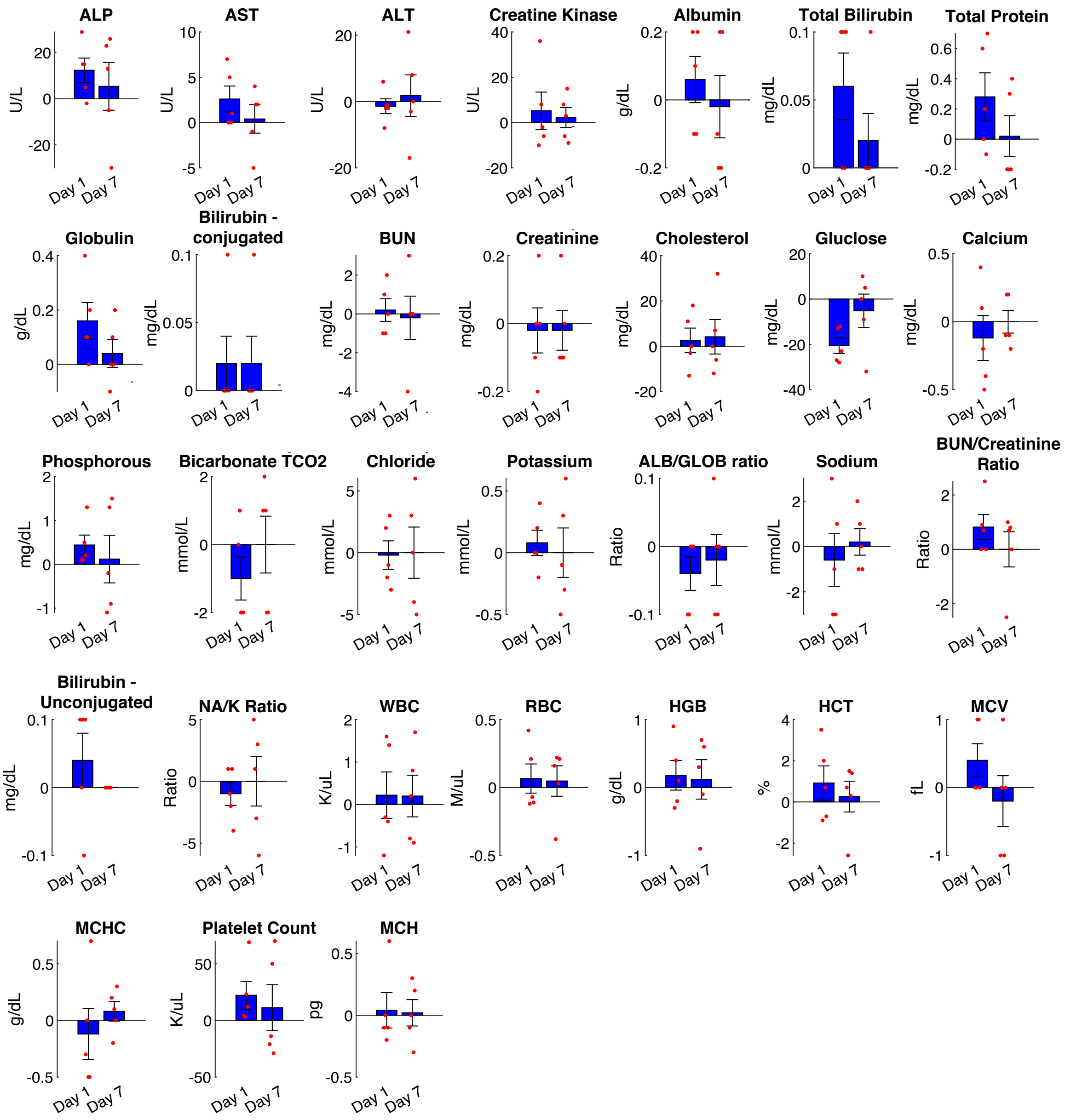

Figure S4. Blood chemistry and hematology values one and seven days after nanodroplet dosing. Change in values, relative to baseline, plotted as individual datapoints (red points) and mean±s.e.m. (blue bars). Results from the five nanodroplet doses at or above 0.5 mg/kg also shown in Fig. 4 and Fig. S3. Only the one-day change in glucose was statistically significant (Table S3; see text for details).

Clinical Chemistry	p-value		95% Confidence Interval	
	Day 1	Day 7	Day 1	Day 7
ALP (U/L)	0.077	0.630	-2.176–26.976	-23.408–34.208
AST (U/L)	0.144	0.811	-1.385–6.585	-3.955–4.755
ALT (U/L)	0.564	0.788	-7.583–4.783	-15.614–19.214
Creatine Kinase (U/L)	0.563	0.645	-17.736–28.136	-10.073–14.473
Albumin (g/dL)	0.426	0.838	-0.128–0.248	-0.274–0.234
Total Bilirubin (mg/dL)	0.070	0.374	-0.008–0.128	-0.036–0.076
Total Protein (mg/dL)	0.154	0.890	-0.162–0.722	-0.357–0.397
Globulin (g/dL)	0.078	0.477	-0.028–0.348	-0.102–0.182
Bilirubin - conjugated (mg/dL)	0.374	0.374	-0.036–0.076	-0.036–0.076
BUN (mg/dL)	0.749	0.866	-1.419–1.819	-3.292–2.892
Creatine (mg/dL)	0.778	0.749	-0.204–0.164	-0.182–0.142
Cholesterol (mg/dL)	0.657	0.611	-12.470–17.670	-16.988–25.388
Gluclose (mg/dL)	**0.004**	0.521	-30.081–-11.119	-25.742–15.342
Calcium (mg/dL)	0.509	1	-0.580–0.340	-0.232–0.232
Phosphorous (mg/dL)	0.125	0.836	-0.191–1.071	-1.392–1.632
Bicarbonate TCO2 (mmol/L)	0.189	1	-2.756–0.756	-2.323–2.323
Chloride (mmol/L)	0.871	1	-3.414–3.014	-5.757–5.757
Potassium (mmol/L)	0.477	1	-0.203–0.363	-0.555–0.555
ALB/GLOB ratio	0.178	0.621	-0.108–0.028	-0.124–0.084
Sodium (mmol/L)	0.634	0.749	-3.838–2.638	-1.419–1.819
BUN/Creatine Ratio	0.148	1	-0.451–2.091	-1.797–1.797
Bilirubin - Unconjugated (mg/dL)	0.374	N/A	-0.071–0.151	0–0
NA/K Ratio	0.351	1	-3.634–1.634	-5.553–5.553
WBC (K/uL)	0.708	0.705	-1.297–1.737	-1.163–1.563
RBC (M/uL)	0.572	0.691	-0.232–0.364	-0.264–0.360
HGB (g/dL)	0.455	0.701	-0.424–0.784	-0.687–0.927
HCT (%)	0.330	0.746	-1.387–3.227	-1.819–2.339
MCV (fL)	0.178	0.621	-0.280–1.080	-1.239–0.839
MCH (pg)	0.794	0.861	-0.358–0.438	-0.276–0.316
MCHC (g/dL)	0.621	0.405	-0.743–0.503	-0.159–0.319
Platelet Count (K/uL)	0.144	0.611	-11.775–56.175	-45.193–67.593

Table S3. Statistical Analysis of nanoparticle blood compatibility. P-values represent the results of one-sample t-tests of the data shown in Fig. S4. $n = 5$ for each test. Bold entries are significant ($p < 0.05$). Confidence intervals indicate the expected range of the change in each value as a result of nanodroplet administration.
